# Effects of flowable liners on the shrinkage vectors of bulk-fill composites

**DOI:** 10.1007/s00784-021-03801-2

**Published:** 2021-01-27

**Authors:** Dalia Kaisarly, D. Meierhofer, M. El Gezawi, P. Rösch, K.H. Kunzelmann

**Affiliations:** 1 Department of Conservative Dentistry and Periodontology, University Hospital, Ludwig-Maximilians-University, Goethestrasse 70, 80336 Munich, Germany; 2 Biomaterials Department, Faculty of Oral and Dental Medicine, Cairo University, Cairo, Egypt; 3 Imam Abdulrahman Bin Faisal University, Dammam, Saudi Arabia; 4 University of Applied Sciences, Augsburg, Germany

**Keywords:** Flowable liner, Bulk-fill composites, Shrinkage vectors, Incremental application, Self-etch adhesive, Medical image registration

## Abstract

**Objectives:**

This investigation evaluated the effect of flowable liners beneath a composite restoration applied via different methods on the pattern of shrinkage vectors.

**Methods:**

Forty molars were divided into five groups (*n* = 8), and cylindrical cavities were prepared and bonded with a self-etch adhesive (AdheSe). Tetric EvoCeram Bulk Fill (TBF) was used as the filling material in all cavities. The flowable liners Tetric EvoFlow Bulk Fill (TEF) and SDR were used to line the cavity floor. In gp1-TBF, the flowable composite was not used. TEF was applied in a thin layer in gp2-fl/TEF + TBF and gp3-fl/TEF + TBFincremental. Two flowable composites with a layer thickness of 2 mm were compared in gp4-fl/TEF + TBF and gp5-fl/SDR + TBF. TEF and SDR were mixed with radiolucent glass beads, while air bubbles inherently present in TBF served as markers. Each material application was scanned twice by micro-computed tomography before and after light curing. Scans were subjected to image segmentation for calculation of the shrinkage vectors.

**Results:**

The absence of a flowable liner resulted in the greatest shrinkage vectors. A thin flowable liner (gp2-fl/TEF + TBFbulk) resulted in larger overall shrinkage vectors for the whole restoration than a thick flowable liner (gp4-fl/TEF + TBF). A thin flowable liner and incremental application (gp3-fl/TEF + TBFincremental) yielded the smallest shrinkage vectors. SDR yielded slightly smaller shrinkage vectors for the whole restoration than that observed in gp4-fl/TEF + TBF.

**Conclusions:**

Thick flowable liner layers had a more pronounced stress-relieving effect than thin layers regardless of the flowable liner type.

**Clinical relevance:**

It is recommended to apply a flowable liner (thin or thick) beneath bulk-fill composites, preferably incrementally.

## Introduction

The polymerization reaction of dental resin composites is always accompanied by polymerization shrinkage, which leads to shrinkage stresses at the cavity boundaries [1]. The gold standard of composite application is incremental application to reduce the C-factor, to compensate for the polymerization shrinkage by the subsequent increment and to improve the degree of conversion [2–4]. The incremental technique is intended to reduce the adverse consequences of polymerization shrinkage. A maximum of a 2-mm-thick increment of conventional composites is essential to assure an adequate degree of polymerization [5, 6].

The application of a flowable liner beneath a composite restoration was introduced when the adhesives were unfilled, i.e., applied in a very thin layer [7, 8]. An intermediate flowable liner reduces the polymerization shrinkage stresses at the bonded interface [9, 10]. In vitro, an intermediate flowable liner below a composite restoration results in an interfacial stress-absorbing layer. This is due to the stress-relieving effect of this elastic flowable composite layer or to its better initial adaptation to cavity boundaries [8, 11].

In contrast, in vivo studies have not detected improved composite restoration performance with an intermediate layer of flowable liner [12–15]. Clinical evaluations are performed by categorization of the restoration according to the FDI criteria, which suggest, among other things, using a probe with a standardized tip diameter for evaluation [16]. Thus, marginal gaps and other differences at a smaller scale could only be quantified by in vitro experiments.

The method of shrinkage vector evaluation is a highly sensitive method that enables the visualization of internal mass movement during polymerization. Micro-computed tomography (micro-CT) scans of composite restorations before and after polymerization allow visualization of the three-dimensional movement of the composite material as vectors with the help of markers, which enables us to understand the amount and direction of polymerization shrinkage [5, 17–23].

Bulk-fill composites have become increasingly popular as a quick or time-saving alternative to the incrementally applied composite restoration materials. However, a shorter restoration time only applies to full-body bulk-fill composites for posterior teeth compared with conventional composites, not flowable bulk-fill composites [24]. Moreover, bulk-fill composites are expected to become an alternative for dental amalgam restorations in preparation for the phase-out of amalgam in Europe by 2030, in accordance with the Minamata Convention on Mercury [25].

There are inconsistent data on the marginal adaptation of bulk-fill composites. Some researchers have found that bulk-fill composites behave similarly to incrementally applied composites [26], while others have found that flowable bulk-fill composites achieve better marginal adaptation than packable bulk-fill composites [27, 28]. The incremental application of a flowable bulk-fill composite reportedly resulted in smaller shrinkage vectors than its bulk application [20].

The aim of this study was to investigate the influence of using a flowable composite liner on the pattern of shrinkage vectors and the adaptation of bulk-fill composites in class I restorations. Moreover, the influence of increasing the thickness of the flowable liner layer and subsequent bulk-fill composite use was studied. The null hypothesis was that the application of a flowable liner does not influence the polymerization shrinkage behavior regardless of the application method.

## Materials and methods

### Sample preparation

Sound extracted human permanent third molars were collected and kept in sodium azide in the dark. After obtaining ethical approval from the committee of the medical faculty of the university (18–360 UE), the experimental procedures were conducted. Forty teeth were divided into five groups (*n* = 8) according to the method of composite application. Cylindrical occlusal cavities 6 mm in diameter and 4 mm in depth were prepared in all teeth. The cavities were prepared at high speed with an air-water coolant. A diamond wheel was used to mark the occlusal circumference of the cavity outline, and then the cavity was prepared with a cylindrical diamond bur. The cavity depth and diameter were checked using a graduated periodontal probe. The use of a cylindrical cavity is in agreement with previous investigations, and it was chosen because the cavity cylindrical configuration exerts no influence [17, 20–23].

All restorations were bonded using a self-etch adhesive (AdheSe, Ivoclar Vivadent, Schaan, Liechtenstein), which was light-cured for 20 s with an LED light-curing device (Bluephase Style, Ivoclar Vivadent, Schaan, Liechtenstein). A universal adhesive was chosen because it is frequently used, the variability of the adhesion is reduced, and the dentin-bonding durability is sufficient for clinical use [29, 30]. The light intensity of the curing light was checked once per week (1100 mW/cm^2^) with a dental radiometer (Bluephase Meter II, Ivoclar Vivadent, Schaan, Liechtenstein). The Bluephase Meter II provides accurate data and is comparable to a laboratory-grade power meter [31].

### Study groups

The materials used in this study are listed in Table 1. The hybrid bulk-fill composite Tetric EvoCeram Bulk Fill (TBF, Ivoclar Vivadent, Schaan, Liechtenstein) was used as the filling material in all cavities. TBF was applied in bulk or in increments, either alone or in combination with a flowable composite as a liner.

**Table 1:** Materials used in this study

Material	Composition	Lot no.	Company
AdheSe Universal (self-etch adhesive)	Methacrylates (67 wt%), water, ethanol (25 wt%), highly dispersed silicon dioxide (4 wt%), initiators, and stabilizers (4 wt%)	W97834	Ivoclar Vivadent, Schaan, Liechtenstein
Tetric EvoFlow Bulk Fill, shade IVA (TEF, flowable bulk-fill composite)	Dimethacrylates (28 wt%), barium glass, ytterbium trifluoride and copolymers (71 wt%), additives, initiators, stabilizers and pigments (<1.0 wt%); total inorganic filler content of 68.2 wt%, inorganic filler particle size ranging between 0.1 μm and 30 μm	W95972	Ivoclar Vivadent, Schaan, Liechtenstein
Tetric EvoCeram Bulk Fill, shade IVA (TBF, hybrid bulk-fill composite)	Dimethacrylates (19.7 wt%), prepolymer (17.0 wt%), barium glass filler, ytterbium trifluoride, mixed oxide (62 wt%), additives, initiators, stabilizers, pigments (< 1.0 wt%)	W93586	Ivoclar Vivadent, Schaan, Liechtenstein
SDR flow+, shade universal (SDR, flowable bulk-fill composite)	Modified urethane dimethacrylate resin, TEGDMA, polymerizable dimethacrylate resin polymerizable trimethacrylate resin, camphorquinone (CQ) photoinitiator, ethyl-4(dimethylamino)benzoate photoaccelerator, butylated hydroxyl toluene (BHT), fluorescent agent and UV stabilizer, fillers (70.5 wt%): barium-alumino-fluoro-borosilicate glass, strontium alumino-fluoro-silicate glass, ytterbium trifluoride glass, silicon dioxide; inorganic filler particle size ranging from 20 nm to 10 μm	1807000856	Dentsply DeTrey GmbH, Konstanz, Germany
Glass beads (added to the flowable composites)	SiO_2_ (72.50 wt%), Na_2_O (13.00 wt%), CaO (9.06 wt%), MgO (4.22 wt%), Al_2_O_3_ (0.58 wt%), diameter: 40–70 μm	Art. no. 5211	Sigmund Lindner GmbH, Warmensteinach, Germany

The flowable bulk-fill composite Tetric EvoFlow Bulk Fill (TEF, Ivoclar Vivadent, Schaan, Liechtenstein) served as the flowable liner that was applied in layers of two different thicknesses (0.5 mm or 2 mm). The thin layer of the flowable liner TEF on the cavity floor was estimated to be 0.5 mm thick, and the 2-mm-thick increment of the flowable liners TEF and SDR was checked both with a graduated periodontal probe before light curing and by the micro-CT scout view before commencing the scanning procedure. The earliest flowable bulk-fill composite, SDR flow+ (SDR, Dentsply DeTrey GmbH, Konstanz, Germany), was applied in one group for comparison with TEF. These two flowable bulk-fill composites have a similar modulus of elasticity [32], while the hybrid bulk-fill composite TBF has a significantly higher modulus of elasticity [33].

**Fig. 1:**
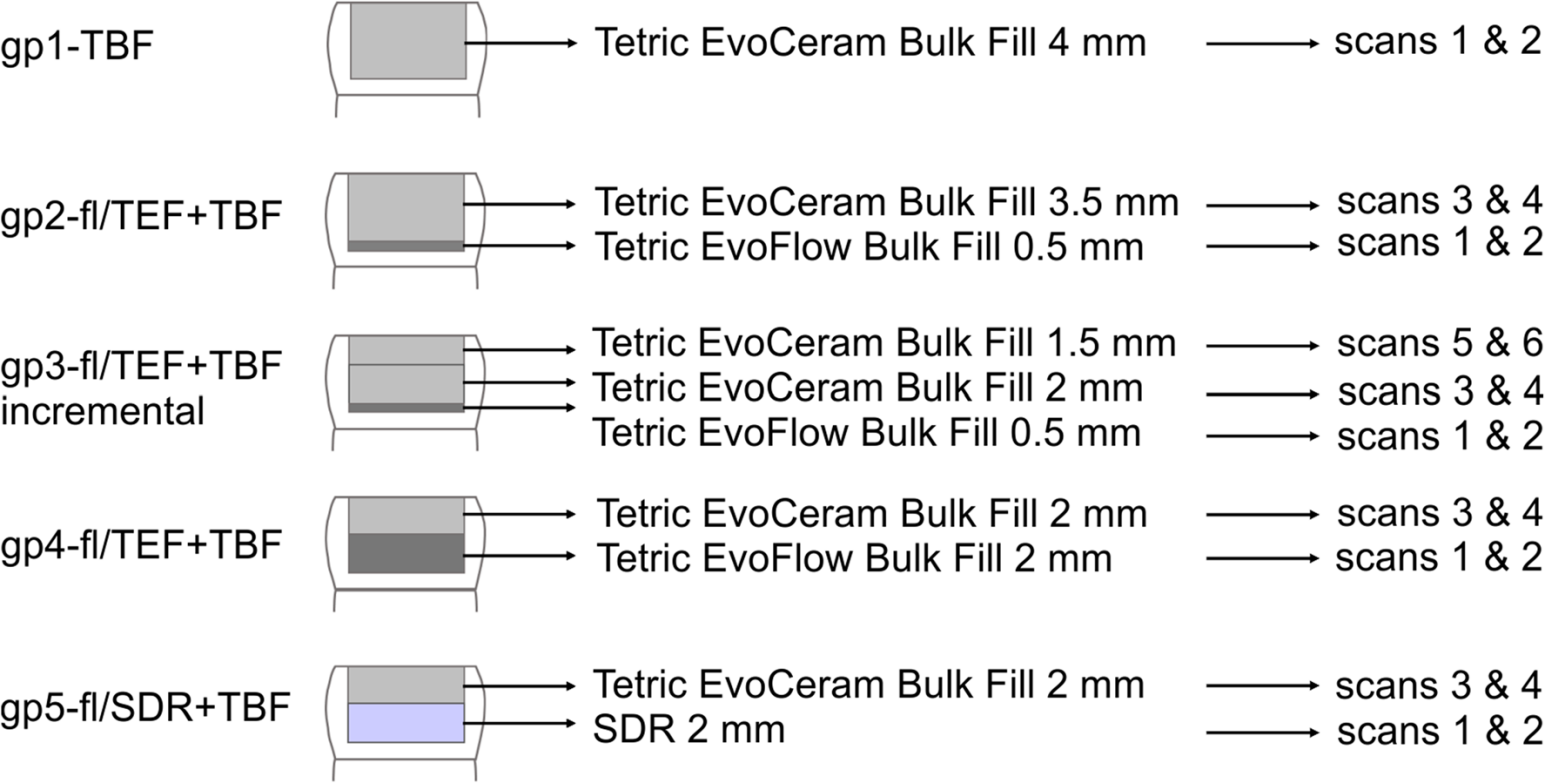
The five study groups with the various applications of a flowable liner and bulk-fill composite and the corresponding micro-CT scans, which were also the combination of micro-CT scans used for data processing

The following groups were designed according to the application method of bulk-fill composites of various viscosities (Fig. 1), and each layer of composite was light-cured for 40 s. In the first group, gp1-TBF, TBF was applied in bulk and served as a control. In the second and third groups, a thin layer (0.5 mm) of the flowable liner was applied, while in the fourth and fifth groups, a 2-mm-thick layer of the flowable liner was applied, assuming that the thicker layer of the flowable liner would stretch or strain more than the thinner layer. The second group, gp2-fl/TEF + TBF, consisted of a thin layer of the flowable liner below TBF applied in bulk application, while the third group, gp3-fl/TEF + TBFincremental, was designed to detect the influence of a thin flowable liner layer and two successive layers of TBF on the shrinkage vectors. The rationale behind studying gp4-fl/TEF + TBF and gp5-fl/SDR + TBF was to measure the two flowable bulk-fill composites TEF and SDR as stress relievers.

### Preparation of traceable composites

The flowable bulk-fill composites TEF and SDR were used, and 2 wt% silanized radiolucent glass beads with an average particle size of 40–70 μm (Sigmund Lindner GmbH, Warmensteinach, Germany) were added to the flowable composites to act as tracer particles [17, 20–23]. The glass beads were chemically bonded to the resin matrix through silanization [34, 35]. The hybrid bulk-fill composite TBF was used in its original status, and small, inherently present air bubbles were used as tracer particles.

### X-ray micro-CT measurements

The samples were scanned by a micro-CT apparatus (Micro-CT 40, Scanco Medical AG, Switzerland) at medium resolution (voxel size, 16 μm) with a cathode current of 114 μA, acceleration voltage of 70 kVp, and integration time of 600 ms. Water was added to the sample holder to prevent dehydration and possible cracking of the tooth during scanning. The sample holder was covered upon scanning with a dark, radiolucent cap to prevent premature polymerization of the uncured composite [17, 20–23]. The flowable composites TEF and SDR were scanned with the average of 1 dataset “average data 1”, while TBF was scanned with the average of 2 datasets “average data 2” to decrease the noise and/or artifacts due to its increased radiopacity.

**Fig. 2:**
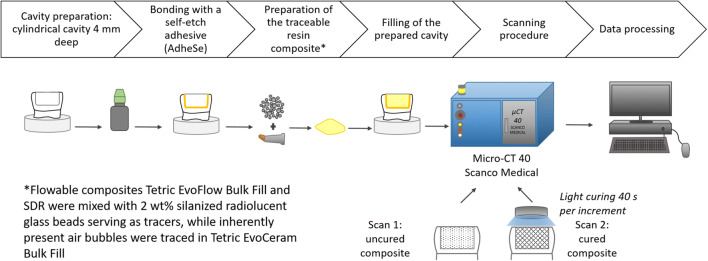
Workflow of this study, including sample preparation, restoration, micro-CT scanning of the samples, and data processing

Each composite sample was scanned in the uncured state and then light-cured for 40 s. The sample was then scanned again in the cured state using the same parameters as before. The raw micro-CT scans were reconstructed and saved as 16-bit datasets of the attenuation coefficient per voxel. The workflow is presented in Fig. 2.

#### Data processing

The first step of the data processing consisted of rigid registration to overlay the prepolymerization scan and the postpolymerization scan. The next step, i.e., sphere segmentation and sphere registration based on the block-matching algorithm, was to identify the embedded glass beads in the flowable bulk-fill composites or the small air bubbles inherently present in the hybrid bulk-fill composite as radiolucent spheres [17, 20–23].

#### Shrinkage vector visualization

The shrinkage vectors of each increment as well as in the whole restoration were visualized three-dimensionally using vtk (www.vtk.org), and each shrinkage vector was represented as a glyph (arrow) pointing in the direction of shrinkage. The shrinkage vectors were scaled by a factor of ten (× 10) for improved visibility, and the shrinkage patterns were analyzed.

#### Shrinkage vector value

The results of the data processing were compiled in a text file where all *x*-, *y*- and *z*-coordinates were listed according to each identified sphere in both the pre- and postpolymerization scans [17, 20–23].

#### Statistical analysis

The mean and standard deviation of the shrinkage vectors in three dimensions and in the axial direction were calculated, tested for normality using the Shapiro-Wilk test and statistically analyzed by one-way ANOVA with Tamhane’s T2 post hoc pairwise comparison using IBM SPSS Statistics 25 [20–23].

#### Scanning electron microscopy

One sample from each group was prepared by longitudinal sectioning and root removal. Then, the sample was cleaned in an ultrasonic water bath for 3 min and left to dry for 24 h. The sample was mounted on a sample holder, sputter-coated with gold, and examined for internal adaptation at a magnification of × 200 at each wall and the cavity floor with a scanning electron microscope (ZEISS GEMINI® FESEM, SUPRA™ 55VP, Carl Zeiss SMT AG, Oberkochen, Germany) [20–23].

## Results

### Shrinkage vector visualization

**Fig. 3:**
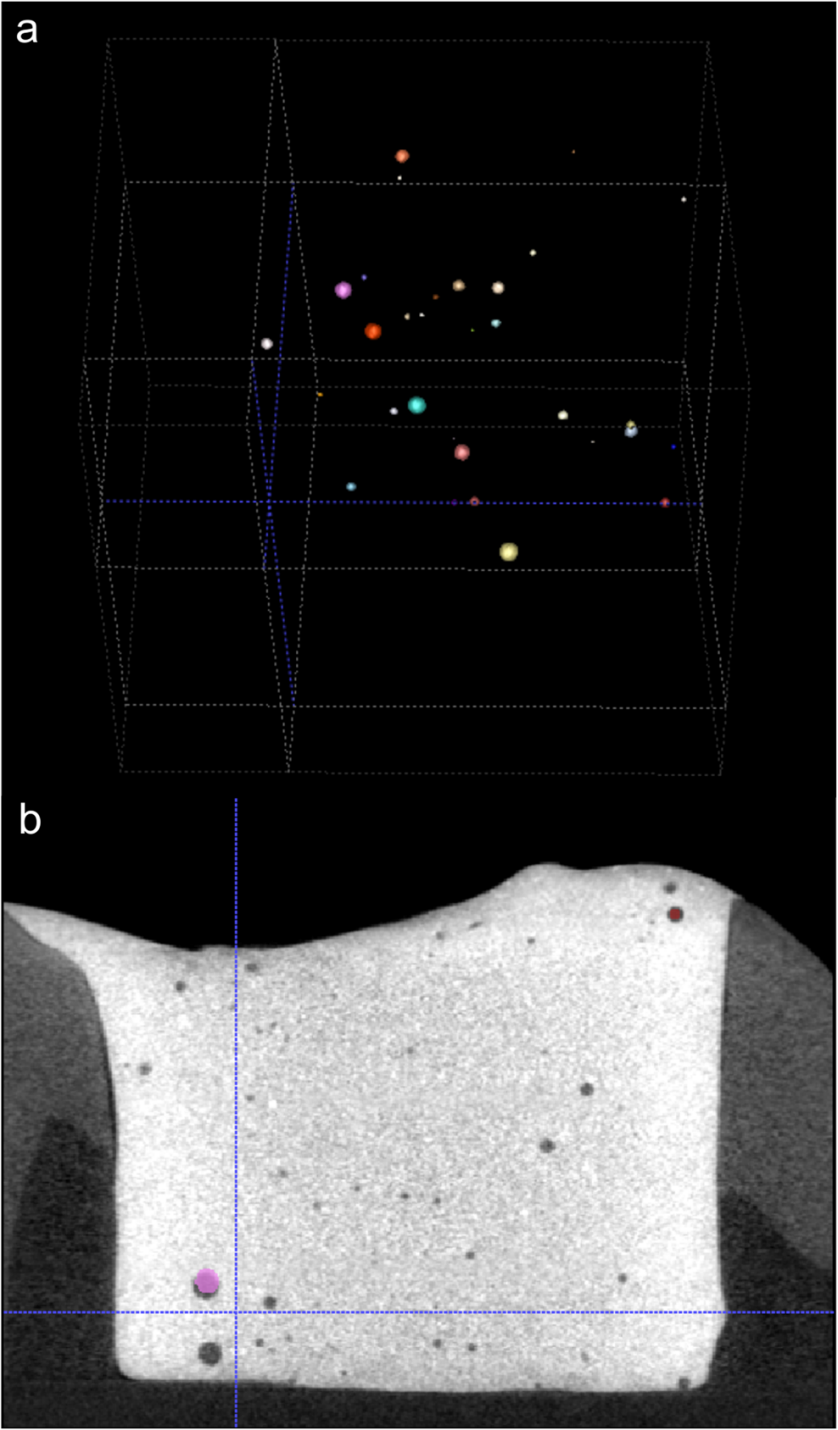
Sphere segmentation and registration. Depending on the tested composite, either embedded glass beads or small, inherently present air bubbles were identified as spheres and extracted in the process of sphere segmentation (a). Sphere registration involves the superimposition of the prepolymerization and postpolymerization scans (b). The spheres in the prepolymerization scan are grayscale, while those in the postpolymerization scan are colored and superimposed on the grayscale spheres. Displacement of the spheres due to polymerization shrinkage is represented by the shrinkage vectors

Image segmentation was performed by sphere segmentation and registration, and the results are shown in Fig. 3. First, the radiolucent spheres that correspond to either the embedded glass beads or the small air bubbles were identified, segmented, and registered in the prepolymerization and postpolymerization scans. Then, the shrinkage vectors were computed and visualized.

**Fig. 4:**
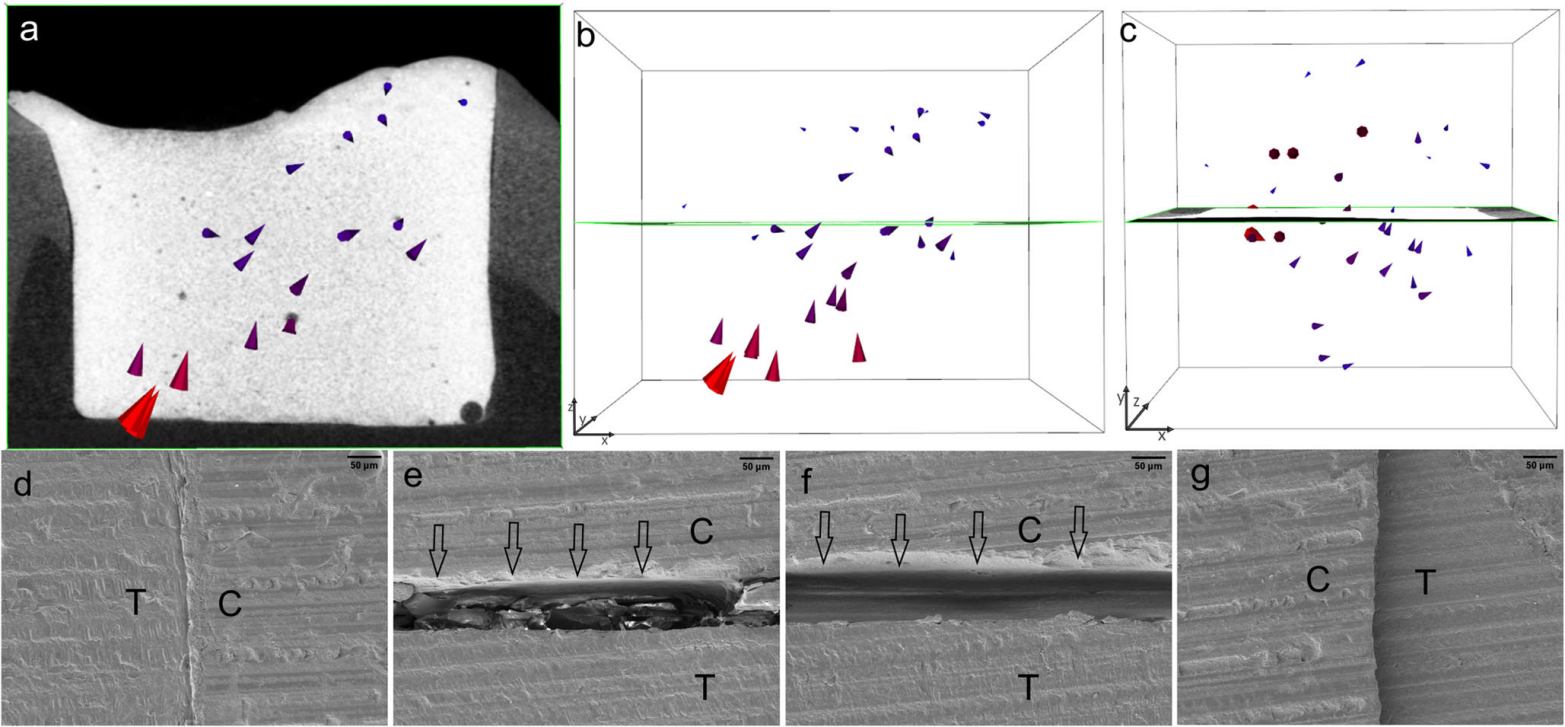
The largest shrinkage vectors pointing upward away from the cavity floor. The bulk application of Tetric EvoCeram Bulk Fill (TBF) in gp1-TBF resulted in very large shrinkage vectors pointing upward and sideward away from the cavity floor (a, b), which can also be visualized from the top view of the restoration (c). The shrinkage vectors are magnified by a factor of 10 for better visualization. The SEM images (at × 200 magnification) display a perfect margin on one side (d) but a large gap (approximately 50 μm) at the cavity floor (e, f)

The shrinkage vector fields consisted of all shrinkage vectors in each sample. The bulk application of TBF in gp1-TBF revealed large shrinkage vectors directed upward and toward one side of the restoration. The restoration floor manifested a large gap, whereas one side showed perfect margins and the other showed only slight detachment from the cavity wall (Fig. 4).

**Fig. 5:**
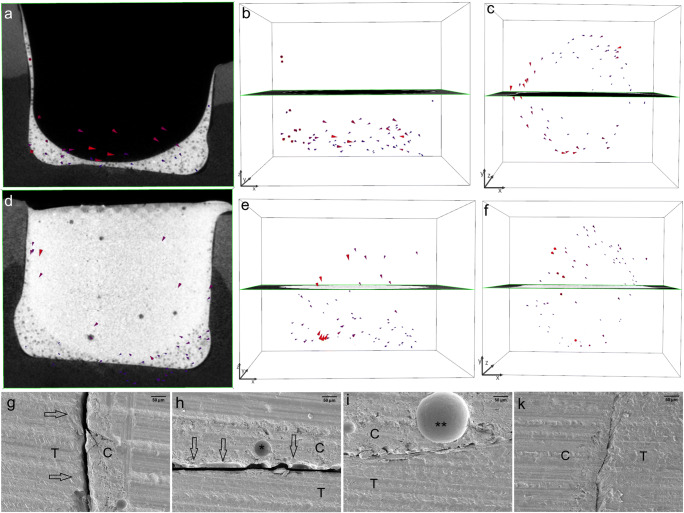
In gp2-fl/TEF + TBF, the shrinkage vectors of the Tetric EvoFlow Bulk Fill (TEF) flowable liner are medium-sized and point toward the right side of the image (a); the radiographic x-plane is located in the background. The unobstructed view of the shrinkage vector field shows the disorder of the shrinkage vectors (b), which appear as a swirl from the top view (c). The layer of flowable liner is conical in shape as the surface tension of the composite drives the material toward the state requiring the least energy. The bulk application of Tetric EvoCeram Bulk Fill (TBF) yields larger shrinkage vectors pointing downward, whereas the lower part, corresponding to the flowable liner, displays smaller shrinkage vectors away from the cavity floor (d, e). From the top view, shrinkage vectors can be observed toward one side of the restoration (f). The shrinkage vectors are magnified by a factor of 10 for better visualization. The SEM images (at × 200 magnification) of one margin (g) and one area of the cavity floor (h) show detachment (arrows) of the restoration from the cavity boundaries, whereas the other areas (i, k) show intimate contact between the restoration and the tooth. The star (*) represents the area of a glass filler, and two stars (**) represent the area of an air bubble within the restoration

In gp2-fl/TEF + TBF, the shrinkage vectors in the thin layer of the TEF flowable liner were directed horizontally within the restoration, similar to a swirl that could be visualized from the top view of the restoration. The flowable liner showed small shrinkage vectors, but the bulk layer displayed larger shrinkage vectors. The SEM images showed debonding on one side of the restoration and in area of the cavity floor, while no debonding was observed in the remaining cavity floor or on the other side of the restoration (Fig. 5).

**Fig. 6:**
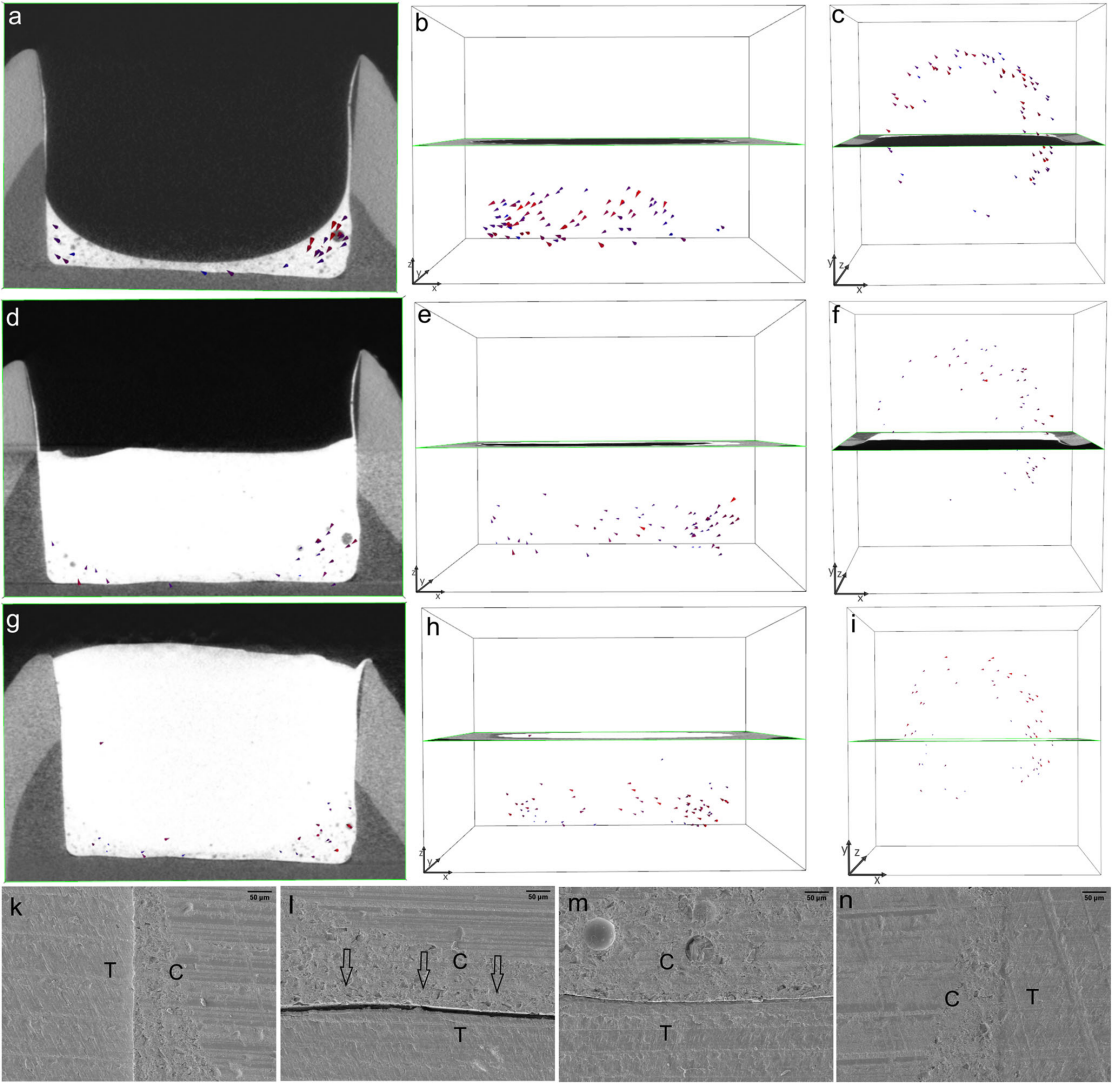
The Tetric EvoFlow Bulk Fill (TEF) flowable liner in gp3-fl/TEF + TBFincremental has many medium-sized shrinkage vectors pointing mainly downward, as seen with the radiographic plane in the background (a, b). The top view reveals shrinkage vectors pointing toward one side of the restoration (c). Increment 1 and increment 2 of Tetric EvoCeram Bulk Fill (TBF) show very few and much smaller shrinkage vectors (d, e, g, h). The radiolucent area on the right side of the cavity is related to the adhesive, which is covered by a thin film of flowable liner (a, d, g). The top view reveals the horizontal movement of shrinkage vectors toward the left side of the cavity (c, f, i). The shrinkage vectors are magnified by a factor of 10 for better visualization. The SEM images (at × 200 magnification) show perfect margins (k, n) and a perfect cavity floor (m) except for a small gap (arrows) in one area of the cavity floor (l)

In the TEF flowable liner in gp3-fl/TEF + TBFincremental, the shrinkage vectors pointed mainly downward, but some small shrinkage vectors close to the cavity floor were directed upward away from the floor. Increment 1 and increment 2 showed very few shrinkage vectors. The restoration showed perfect margins in the SEM images, and only one area of the cavity floor displayed some detachment (Fig. 6).

**Fig. 7:**
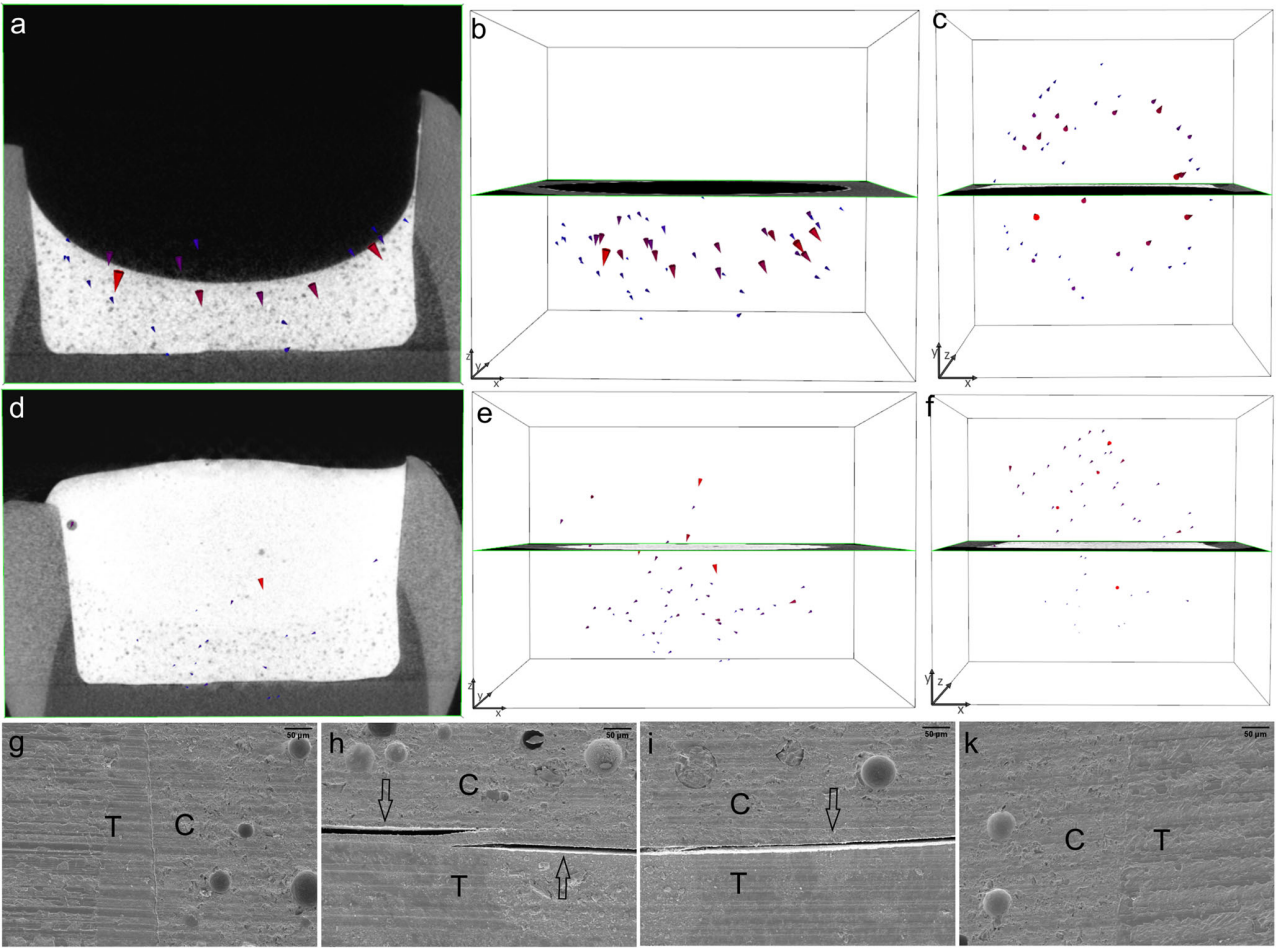
In gp4-fl/TEF + TBF, the 2-mm-thick layer of the Tetric EvoFlow Bulk Fill (TEF) flowable liner has numerous large shrinkage vectors pointing toward the cavity floor, with the radiographic plane in the background (a, b). The top view reveals many shrinkage vectors pointing toward one side of the restoration (c). The second increment of Tetric EvoCeram Bulk Fill (TBF) displays small shrinkage vectors pointing downward toward the cavity floor (d, e). From the top view, the shrinkage vectors are directed toward the opposite side compared with those in the flowable liner layer (f). The shrinkage vectors are magnified by a factor of 10 for better visualization. The SEM images (at × 200 magnification) show perfect margins (g, k) but detachment from the cavity floor (h, i), where the detachment is between the TEF and the adhesive as well as between the adhesive and the dentin (h)

In gp4-fl/TEF + TBF, the shrinkage vectors in the thick layer of the TEF flowable liner were directed downward toward the cavity floor. The covering/capping layer of TBF also displayed downward movement of the free surface, and the previously cured flowable liner exhibited numerous small shrinkage vectors also pointing toward the cavity floor. The margins of the vertical walls were perfect. However, the composite detached from the cavity floor; the adhesive failure was between the composite and the adhesive in one area and between the adhesive and the dentin in another area (Fig. 7).

**Fig. 8:**
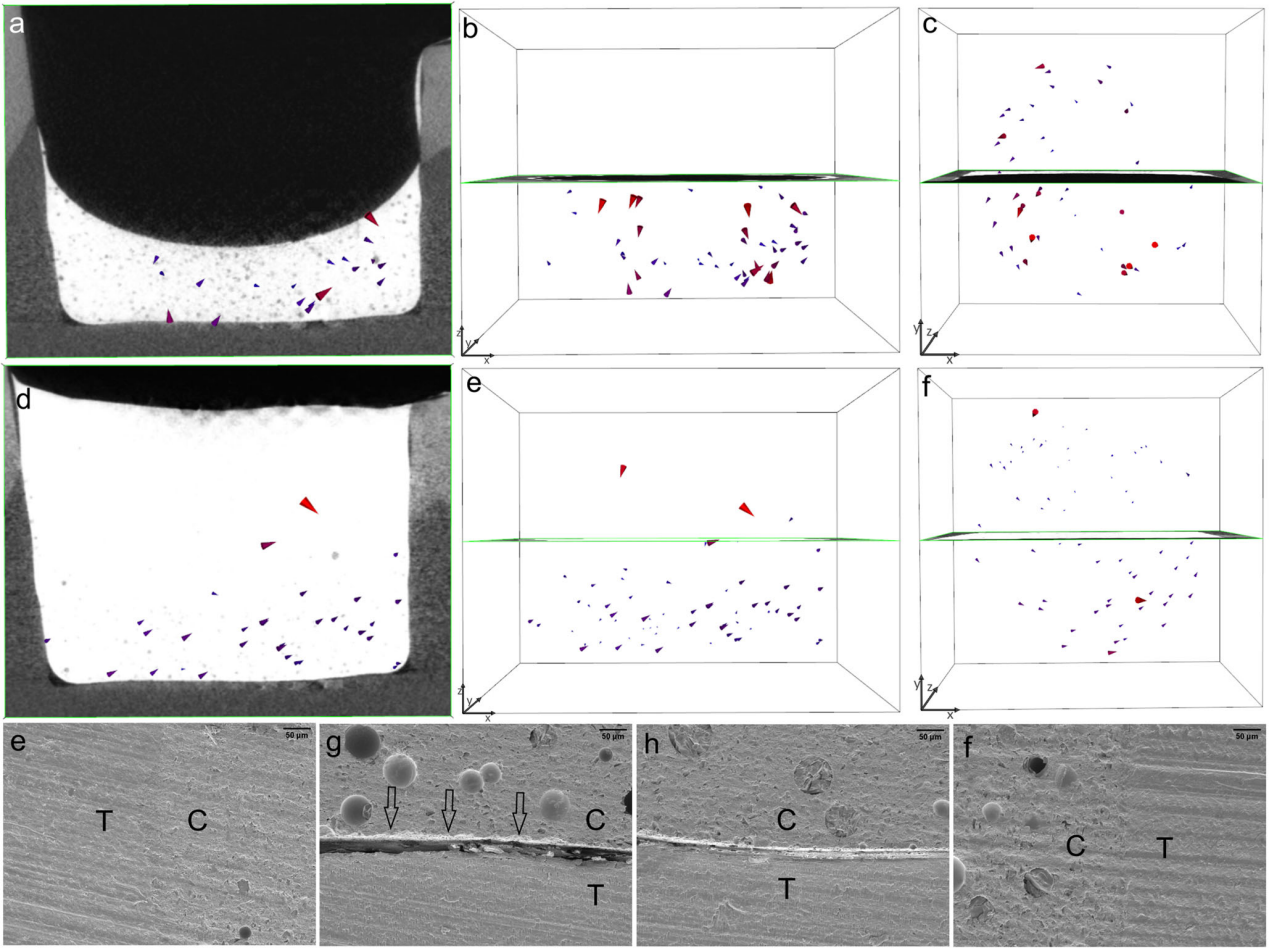
In gp5-fl/SDR + TBF, the shrinkage vector field in the SDR flowable liner is irregular (a, b), and the shrinkage vectors are arranged in a swirl as seen in the top view (c). The covering increment of Tetric EvoCeram Bulk Fill (TBF) has few shrinkage vectors pointing downward, whereas the shrinkage vectors in the flowable liner point upward and sideward (d, e), also in the form of a swirl (f). The shrinkage vectors are magnified by a factor of 10 for better visualization. The SEM images (at × 200 magnification) show perfect margins (e, f) but gaps of variable width at the cavity floor (g, h)

In gp5-fl/SDR + TBF, the thick layer of the SDR flowable liner showed a random shrinkage vector field, where some vectors at the free surface pointed downward, others in close proximity to the cavity floor pointed upward, and others were directed sideward. In the covering layer, many shrinkage vectors pointed upward and sideward toward one side of the restoration. The SEM images displayed perfect vertical cavity margins and detachment, to various extents, at the cavity floor (Fig. 8).

### Shrinkage vector value

The shrinkage vector data were not normally distributed (*p* < 0.05). However, according to Winer et al., one-way ANOVA requires data with only a nearly normal distribution because it is robust to violations of normality and can still provide valid results [20, 36].

#### Quantitative nondirectional analysis

The absence of a flowable liner resulted in greater shrinkage vectors compared to the presence of a thin underlying flowable liner. In gp2-fl/TEF + TBFbulk, the thin flowable liner resulted in larger overall shrinkage vectors for the whole restoration than the thick flowable liner in gp4-fl/TEF + TBF. The thin flowable liner and incremental composite application in gp3-fl/TEF + TBFincremental yielded the smallest shrinkage vectors. SDR as a flowable liner yielded slightly smaller shrinkage vectors for the whole restoration in gp5-fl/SDR + TBF than in gp4-fl/TEF + TBF. The results of the mean vectors are listed in Table 2. One-way ANOVA revealed a significant difference (F = 33.772; Df = 94,454; *p* < 0.001), and the post hoc pairwise comparison using Tamhane’s T2 test showed significant differences among the groups.

**Table 2:** Shrinkage vectors (mean **±** standard deviation; μm)

Group	Group with successive increment(s)	Shrinkage vector (mean ± standard deviation; μm)	Shrinkage vector on z-axis (mean ± standard deviation; μm)*
Group 1	Gp1-TBF	37.1 ± 31.0 (a)	1.1 ± 39.0 (a, b, c, d, e, f)
Group 2	Gp2-fl/TEF	15.1 ± 7.2 (f, g, h)	− 0.6 ± 8.4 (a, c, d, f)
	Gp2-fl/TEF + TBF	24.0 ± 18.8 (b, c, d, e)	8.8 ± 22.1 (b, e)
Group 3	Gp3-fl/TEF	16.4 ± 6.3 (d, f, g, h)	0.2 ± 9.2 (a, c, f)
	Gp3-fl/TEF + TBFinc1	12.5 ± 6.7 (i)	− 0.3 ± 7.1 (a, c, d, f)
	Gp3-fl/TEF + TBFinc1 + TBFinc2	13.4 ± 6.0 (i)	− 1.6 ± 7.7 (a, d, f)
Group 4	Gp4-fl/TEF	21.5 ± 21.6 (b, c, d, e, f)	6.4 ± 16.6 (b, e, f)
	Gp4-fl/TEF + TBF	20.6 ± 33.6 (b, c, d, e, f, g)	1.3 ± 38.1 (a, c, d, e, f)
Group 5	Gp5-fl/SDR	24.2 ± 23.1 (b, c, d, e)	1.8 ± 18.3 (a, c, f)
	Gp5-fl/SDR + TBF	18.1 ± 26.7 (c, d, f, g, h)	− 1.2 ± 23.0 (a, c, d, f)

Different letters indicate statistically significant differences between the groups within one column. * Negative values represent upward movement toward the light source, whereas positive values denote downward movement toward the cavity floor

#### Quantitative directional analysis

The directional analysis investigated the *z*-component of the 3D shrinkage vectors to separately evaluate the movement along the *z*-axis. Negative values represent upward movement toward the light source, whereas positive values denote movement toward the cavity floor. The results of the axial movement are listed in Table 2.

The greatest downward shrinkage was seen in gp2-fl/TEF + TBF and gp4-fl/TEF + TBF, followed by slight downward movement in gp5-fl/SDR + TBF, gp4-fl/TEF + TBF, gp1-TBF, and gp3-fl/TEF. Upward movement was observed in gp3-fl/TEF + TBFinc1 + TBFinc2, gp5-fl/SDR + TBF, gp2-fl/TEF, and gp3-fl/TEF + TBFinc1. One-way ANOVA revealed a significant difference (F = 11.902; Df = 94,520; *p* < 0.001), and the post hoc pairwise comparison using Tamhane’s T2 test showed significant differences among the groups.

## Discussion

The null hypothesis can be rejected because the polymerization shrinkage behavior of the applied composites was affected by the presence of a flowable liner and varied with the application method. The shrinkage vector evaluation displayed greater shrinkage vectors in the bulk application than in the horizontal incremental application. Moreover, thicker layers of flowable liner resulted in smaller shrinkage vectors than thinner layers of flowable liner, except when the covering composite was also applied in increments.

Our findings show that when a flowable liner was applied, smaller shrinkage vectors were obtained in the flowable liner and the following increment(s) which might be related to the relative elasticity of the intermediate flowable composite layer. The largest divergence among the shrinkage vector values was identified between the bulk application of composite without any flowable liner and the application of composite with a flowable liner, which is a logical finding related to the volume of the inserted composite and the existence of an elastic intermediate zone of flowable composite. The presence of an intermediate layer of flowable liner mainly influenced the magnitude and, to a certain extent, the direction of the shrinkage vectors.

In our study, the flowable liner could not be applied as a uniformly thick layer and varied in thickness. Cavity corners or line angles hosted a larger volume of the flowable liner that was cone-shaped, especially when applied in thin increments. A similar phenomenon was observed when the adhesive layer was increased in thickness [37]. Moreover, the thick layer of flowable liner had a concave surface because the composite was well adapted to the cavity boundaries due to capillary action. This is in agreement with an earlier observation of SDR when applied in bulk versus in increments [20]. The intimate adaptation of flowable composites due to decreased viscosity has previously been hypothesized [8, 11, 38]. The thin layer of flowable liner showed smaller shrinkage vectors than did the thick layer, which could be related to the greater volume in the thicker increment. This is in agreement with the observation that axial shrinkage stress depends on the C-factor as well as on the composite mass [39, 40].

The bulk application of composite without a flowable liner in gp1-TBF resulted in the largest shrinkage vectors but, surprisingly, only small axial movement downward toward the cavity floor. This phenomenon could be explained by internal mass movement in various areas within the restoration, such as downward movement of the free surface, as well as upward and horizontal movement, possibly resulting in shear forces that could lead to a large gap at the cavity floor, as displayed in the SEM images. This observation is in agreement with the results of an evaluation of shrinkage vectors in a nonbonded cavity, where the shrinkage was directed toward the center of the restoration, and almost no axial movement was detected (0.5 μm) in relation to the mean shrinkage vector of 23.5 μm [22].

The incremental application of the bulk-fill composite in gp3-fl/TEF + TBFincremental above the flowable liner resulted in even smaller shrinkage vectors and the best adaptation to the cavity boundaries. Although the composite above the flowable liner in gp2-fl/TEF + TBF showed favorable axial movement downward toward the cavity floor, the SEM images showed detachment from the cavity margins and the cavity floor. Comparing the thicker increments of flowable liner (gp4-fl/TEF + TBF and gp5-fl/SDR + TBF) revealed slightly smaller shrinkage vectors in the TEF than in the SDR, which was also reflected in the axial movement and SEM images.

The axial movement, or the movement of the z-component of the shrinkage vectors, was found to be limited. The maximum upward movement away from the cavity floor was 1.6 μm, which is less than that measured amount of upward movement in previous investigations (4.5–29.1 μm) [19–21, 23]. Greater axial movement downward toward the cavity floor was measured in the bulk-fill group with a thin flowable liner (gp2-fl/TEF + TBF) and in the group with a thick layer (2 mm) of flowable liner (gp4-fl/TEF + TBF).

Some researchers have advocated the bulk placement of bulk-fill composites to provide relatively gap-free tooth-restoration interfaces [41], while others have found that bulk-fill composites perform similar to conventional composites in the clinic [42]. However, our results did not confirm these observations.

The results of the current investigation advocate the use of a flowable liner as well as the incremental placement of bulk-fill composites, depending on the cavity depth. This is in line with previous reports showing that incrementally applied composites exhibit better internal adaptation than those applied in bulk [20, 43]. Moreover, these results are consistent with earlier conclusions that the efficacy of bonding to the cavity bottom depends on the C-factor and the type of bulk-fill composite used and that flowable bulk-fill composites exhibit satisfactory bond strength values [44, 45].

The incremental application is important for densification, adaptation, and bond formation/strength [46, 47]. Moreover, not only the filling technique but also the cavity size is a decisive factor in class I cavities [19, 48]. The use of a 2-mm-thick flowable liner rather than thinner increments has been recommended for less marginal leakage [49]. Based on our results, we can recommend the application of both thin and 2-mm-thick layers of flowable liner for a favorable outcome; however, a thick layer of flowable liner is slightly more favorable.

Universal adhesives can be used in self-etch mode or in combination with a phosphoric acid etchant in total-etch mode [50]. In the current investigation, a universal adhesive was applied without prior acid etching in self-etch mode. This application yielded only a few marginal gaps when the composites were applied in increments. The thickness and rigidity of the adhesive layer are important properties regarding the mechanical behavior of the restored tooth and thus play important roles in attenuating the polymerization and occlusal stresses [7, 51].

Although SEM images of only one sample per group were obtained, they displayed gaps and debonding at the interface between the tooth and the restoration. Even if the adaptation between the cavity walls and the restoration was perfect in many instances, the debonding at the cavity floor was variable and increased in width with increases in the volume of composite applied at a time, such as in the bulk application. However, sample sectioning, sample overdrying, and/or the effect of the high vacuum needed for SEM observation might also lead to gap formation [52]. Using micro-CT scans of composite restorations for the nondestructive evaluation of interfacial gaps can overcome the aforementioned limitations [53].

Extended light curing of 40 s was performed in the current study to ensure sufficient curing, to overcome possible variations in the light beam intensity, and to compensate for involuntary changes in position during curing [54, 55]. Moreover, light curing through the thin layer of flowable liner could help in the conversion of the oxygen-inhibited layer of the adhesive, improving the bond strength [56].

The prepared cavity in the current study was a cylindrical class I cavity with an unfavorable C-factor; many studies evaluating flowable liners have been conducted in class II cavities, in which the marginal integrity of the cervical margin is an important aspect [12–15, 57–61]. Earlier in vivo investigations of flowable liners did not detect any improvement in postoperative hypersensitivity or marginal adaptation [12–15, 62]. In contrast, in vitro studies revealed improved performance of restorations with underlying flowable liners [7, 8, 10, 51].

Previous studies found that flowable liners did not reduce polymerization shrinkage stresses [63, 64]. However, current formulations of bulk-fill composites incorporate modifications to decrease shrinkage stresses by a shrinkage stress reliever in TBF and a stress modulator in SDR, which acts as a spring and reduces stresses within the restoration [33, 65]. In the current study, SDR was used in only one group in a 2-mm-thick increment. Studying the use of thin increments of SDR as a flowable liner might have given broader information on how it performs below a hybrid bulk-fill composite. However, SDR was included in the current study as a reference material since it is a well-established and commonly used flowable bulk-fill composite. The volumetric shrinkage and shrinkage vectors of this material (bulk versus incremental application) have been investigated before [20, 66, 67]. Due to its decreased shrinkage stress and relatively low volumetric shrinkage compared with other composites, including TEF, SDR was only used in a 2-mm-thick increment for the best use of these reported advantages [32, 68].

The incorporation of glass beads into the flowable composites slightly altered their viscosity and might affect their polymerization kinetics, which was not investigated in the current study. The amount of glass beads incorporated in the flowable composites was standardized (2 wt%), and without them, the mass movement upon polymerization could not have been traced [20–23]. However, the bulk-fill composite TBF has a high viscosity, hindering the incorporation of glass spheres. More importantly, due to the heterogeneous radiographic appearance of TBF, the software was unable to identify the incorporated glass beads as spheres and thus could not compute the shrinkage vectors. In our study, the small, inherently present air bubbles served as tracer particles, while Takemura et al. deliberately incorporated air bubbles into the composite [69].

The limitations of the shrinkage vector evaluation method include the need to incorporate tracer particles, such as radiolucent glass beads or radiopaque zirconia fillers, to trace the mass movement upon polymerization [17–23] unless inherently present structural components can be traced [19]. Furthermore, the radiopacity of the investigated composites is another important factor that is influenced by the composition. TBF has a high radiopacity, which results in artifacts and might interfere with the micro-CT-based shrinkage vector evaluation. Thus, in our case, we could overcome the noise by scanning with “average data 2,” which means that double the number of projections was performed, and a mean value was obtained. Furthermore, in contrast to TEF and SDR, TBF has a heterogeneous radiographic appearance that interferes with the identification of the embedded radiolucent glass beads as spheres [17, 20–23]. Tooth-restoration interfacial gaps were not quantified in the current investigation, although they would provide further information on possible debonding from the cavity boundaries due to increased shrinkage stresses [19]. Conventional methods for evaluating polymerization shrinkage involve in vitro methods, such as microleakage testing, SEM examination of internal adaptation, linear or volumetric polymerization shrinkage evaluation, or cuspal deflection testing, or in vivo methods, such as the evaluation of fillings according to the FDI criteria or the resin replica technique [5, 16, 70, 71].

The method of shrinkage vector evaluation is a highly sensitive and accurate method that allows for the visualization and detection of shrinkage vectors due to mass movement upon polymerization. The nondestructive method of testing based on micro-CT enables visualization of the internal movement of not only composites on a micrometer scale but also the related interfaces, which could not be seen otherwise. Our research provides evidence to help dental clinicians in decision-making during their daily practice of restorative dentistry.

## Conclusions

The application of a flowable liner and varying the thickness of the applied flowable liner and the covering composite influenced the magnitude of the shrinkage vectors. The thinner the increment of the flowable liner or the supervening composite, the smaller the magnitude of the shrinkage vectors. The smaller shrinkage vectors in the covering increment(s) were influenced by the shrinkage vectors of the previously cured increment(s). Both thin (0.5 mm) and thick (2 mm) layers of flowable liner led to favorable shrinkage patterns and adaptation to the cavity boundaries. Thus, it can be concluded that flowable liners act as a stress reliever. It is recommended to apply a thin or thick layer of flowable liner beneath bulk-fill composites, preferably incrementally.
